# The complete mitochondrial genome of *Moolgarda perusii* (Teleostei: Mugilidae)

**DOI:** 10.1080/23802359.2020.1847609

**Published:** 2021-01-13

**Authors:** Yi Liu, Yexin Yang, Hongmei Song, Chao Liu, Xuejie Wang, Yinchang Hu, Xidong Mu

**Affiliations:** a Key Laboratory of Leisure Fisheries, Ministry of Agriculture and Rural Affairs, Guangdong Modern Recreational Fisheries Engineering Technology Center, Pearl River Fisheries Research Institute, Chinese Academy of Fishery Sciences, Guangzhou, China;; b Key Laboratory of Aquatic Animal Immune Technology of Guangdong Province, Guangzhou, China

**Keywords:** Mitochondrial genome, Mugilidae, *Moolgarda perusii*

## Abstract

We determined the complete mitochondrial genome of *Moolgarda perusii*, which is 16,781 bp in length, and contains 13 protein-coding genes (PCGs), two rRNAs, 22 tRNAs, and a complete control region. The total base composition of the mitogenome is 28.7% T, 27.0% C, 28.5% A, and 15.9% G. Of the 13 PCGs, 11 PCGs start with an ATG codon. Eight PCGs use TAA/TAG/AGA as the termination codon, whereas five PCGs have incomplete stop codon TA/T. This study would be useful for further studying population genetics and understanding the phylogenetic relationship of the family Mugilidae.

*Moolgarda perusii* belonging to the family Mugilidae is widely distributed in Indo-West Pacific: Africa to the Mariana Islands. Members of family Mugilidae are widespread across freshwater, estuarine, and marine habitats of tropical and temperate latitudes. There are a few studies on mitochondrial (mt) genome of family Mugilidae (Miya et al. 2001; Durand et al. 2012). Hence, we sequenced its mt genome of *M. perusii* and analyzed its phylogenetic position in order to provide data for the studies on population genetic diversity, genetic relationship of Mugilidae.

In this study, a newly complete mt genome of *M. perusii* collected from Guangdong Lanhai Marine Technology Co., Ltd., Guangdong Province of China (23°12′51″N, 113°28′06″E) was determined. The voucher specimen (no. MU-MP-1) is deposited at National Freshwater Aquatic Germplasm Resource Center in Guangzhou city, Guangdong Province in China. The total genomic DNA was extracted using the Tissue DNA Kit (OMEGA E.Z.N.A., Doraville, GA) following the manufacturer’s protocol. The DNA was then subjected to conduct next-generation sequencing which generated 20 GB raw pairend reads (150 bp PE sequencing on Illumina Nova6000). The mitogenome genome was assembled with SPAdes v.3.5.0 software (http://cab.spbu.ru/software/spades/) (Lapidus et al. 2014).

The mt genome sequence of *M. perusii* is 16,781 bp (28.5% A, 28.7% T, 15.9% G, and 27.0% C) in length and contains 13 protein-coding genes (PCGs), 22 tRNA genes, and two rRNA genes and a control region (CR). The gene arrangement and orientation are identical with the *Liza haematocheila* from the same family. Of the 13 PCGs, 11 PCGs start with ATG, except ND1 and COX I starting with GTG; eight PCGs use the stop codon TAA/TAG/AGA, whereas five PCGs (COX2, *ATP*ase6, COX3, ND4, and Cyt *b*) stop with the incomplete codon T and TA.

Among the 13 PCGs ranging in size from 174 bp (*ATP*ase8) to 1863 bp (ND5), 12 PCGs are coded on the H-strand, while ND6 is coded on the L-strand. The 12S and 16S rDNA are 950 bp and 1730 bp in length, respectively. The length variations of 22 tRNAs range from 66 bp (tRNA^Cys^) to 75 bp (tRNA^Lys^). There are five non-coding regions: the L-strand replication origin region (32 bp) locating between tRNA^Asn^ and tRNA^Cys^, the 86 bp region between *ATP*ase8 and *ATP*ase6, the 35 bp region between Cyt *b* and tRNA^Thr^, the 34 bp region between tRNA^Thr^ and tRNA^Pro^, and the CR (858 bp) locating between tRNA^Pro^ and tRNA^Phe^.

To determine taxonomic status of *M. perusii,* the phylogenetic relationship was reconstructed using Bayesian inference (BI) methods based on the nucleotide sequence of 13 PCGs (Figure 1). The result supported the monophyly of the family Mugilidae, consistent with previous studies (Miya et al. 2001; Durand et al. 2012), and showed that *M. perusii* was clustered to the *M. cunnesius* with high bootstrap values (100%). The complete mt genome sequence of *M. perusii* would be useful for further studying population genetics and understanding the phylogenetic relationship of the family Mugilidae.

**Figure 1.**
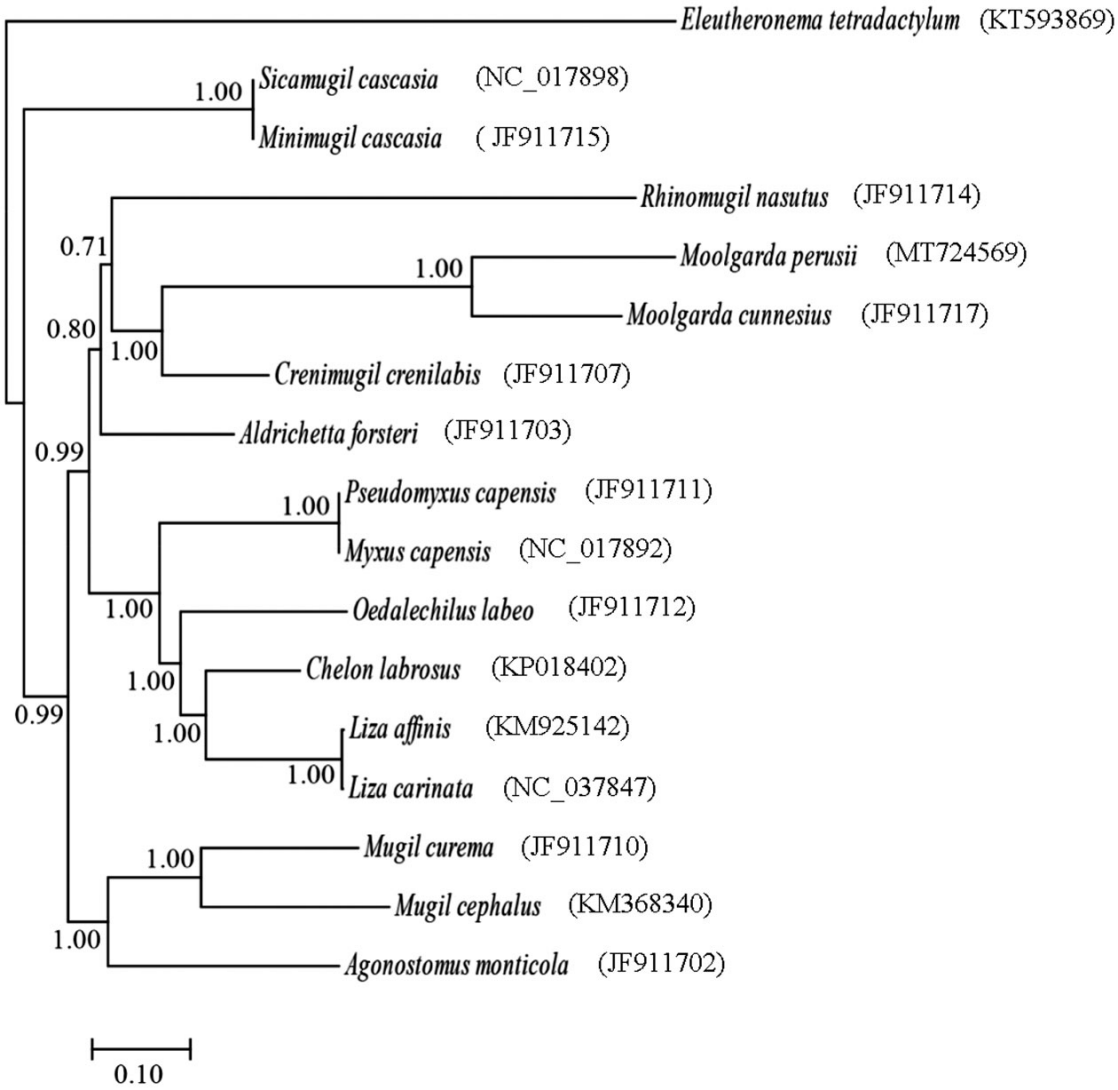
The phylogenetic relationships of the family Mugilidae based on the nucleotide sequence of 13 protein-coding genes in the mitochondrial genome. The tree was constructed based on Bayesian inference (BI) methods using MrBayes software (Ronquist and Huelsenbeck 2003). The *Eleutheronema tetradactylum* (KT593869) was used as outgroup.

## Data Availability

The data that support the findings of this study are openly available in GenBank at https://www.ncbi.nlm.nih.gov/genbank/, accession number MT724569.

## References

[CIT0001] Durand JD, Chen WJ, Shen KN, Fu C, Borsa P. 2012. Genus-level taxonomic changes implied by the mitochondrial phylogeny of grey mullets (Teleostei: Mugilidae). C R Biol. 335(10–11):687–697. 23199637 10.1016/j.crvi.2012.09.005

[CIT0002] Lapidus A, Antipov D, Bankevich A, Gurevich A, Korobeynikov A, Nurk S, Prjibelski A, Safonova Y, Vasilinetc I, Pevzner PA. 2014. New frontiers of genome assembly with SPAdes 3.0 (poster).

[CIT0003] Miya M, Kawaguchi A, Nishida M. 2001. Mitogenomic exploration of higher teleostean phylogenies: a case study for moderate-scale evolutionary genomics with 38 newly determined complete mitochondrial DNA sequences. Mol Biol Evol. 18(11):1993–2009. 11606696 10.1093/oxfordjournals.molbev.a003741

[CIT0004] Ronquist F, Huelsenbeck JP. 2003. MrBayes 3: Bayesian phylogenetic inference under mixed models. Bioinformatics. 19(12):1572–1574. 12912839 10.1093/bioinformatics/btg180

